# The Implementation of Renal Denervation in the Management of Resistant Hypertension Despite Use of Multitherapy Antihypertensives at Maximally Tolerated Doses: A Contemporary Literature Review

**DOI:** 10.7759/cureus.41598

**Published:** 2023-07-09

**Authors:** Mukosolu F Obi, Manjari Sharma, Maria Andrea Reinberg, Zola N'Dandu, Cho Hyun Joon, Melissa Vega

**Affiliations:** 1 Internal Medicine, Wyckoff Heights Medical Center, Brooklyn, USA; 2 Internal Medicine, St. George's University, True Blue, GRD; 3 Interventional Cardiology, Ochsner Medical Center, New Orleans, USA

**Keywords:** radiance-htn trio trial, cardiovascular disease, clinical trial, treatment resistant hypertension, spyral htn-off med trial, spyral htn-on med trial, simplicity trials, renal denervation therapy

## Abstract

Refractory hypertension is highly prevalent among the hypertensive population, and current clinical management has failed to provide optimal control for these individuals. This subtype of arterial hypertension is defined as a persistently elevated systolic blood pressure reading of 140 mmHg, or higher, despite multiple antihypertensive use at maximally tolerated dosing. These patients have an elevated risk of cardiovascular and renal complications, urging for the need of more effective therapeutic management. Renal sympathetic efferent nerves have been noted to play an important role in volume and blood pressure homeostasis. Before the implementation of oral antihypertensives, the use of surgical lumbar sympathectomy for the reduction of persistent hypertension was considered a life-saving approach. However, individuals were left with debilitating side effects, such as postural hypotension, syncope, and impotence. A new and minimally invasive technique has been proposed, where the kidneys undergo selective denervation in hopes of providing decreased cardiovascular morbidity and mortality for patients with resistant hypertension. Some studies demonstrated promising outcomes with a reduction in blood pressure, a decrease in medication reliance, and a potential long-lasting effect of the procedure with an overall improvement in cardiovascular health. Unfortunately, most of the available data was obtained from observational, uncontrolled studies with short-term follow-up, small sample sizes, and high variability in blood pressure measurement. Therefore, further evidence is needed to determine whether renal denervation provides long-term benefits for blood pressure control and improves outcomes for mortality and cardiovascular events in this patient population.

## Introduction and background

Hypertension (HTN) has an increasing predominance worldwide, with refractory HTN having a prevalence of at least 10%-20% among the hypertensive population [1,2]. Renal sympathetic hyperactivity has been identified as the major cause of resistant hypertension, and thus, renal sympathetic ablation has been proposed as a possible therapeutic alternative to this condition. The ineffectiveness of various interventions, including dietary and lifestyle changes, alongside medication, highlights the need for new, safe, and efficient treatments for this condition. Resistant or refractory hypertension is defined as persistently elevated high blood pressure (BP) despite adherence to at least three different classes of antihypertensives at maximally tolerated doses, including a diuretic [1,2]. The catheter-based renal denervation (RDN) technique emerges as a promising and innovative treatment approach for refractory hypertension. In the past, surgical lumbar sympathectomy was employed during the 1950s as a treatment for resistant hypertension. However, this procedure was eventually abandoned due to various complications, including postural hypotension, syncope, and impotence [3]. The renal sympathetic efferent nerves play a vital role in regulating volume expansion and maintaining blood pressure equilibrium by innervating the renal tubules, vasculature, and juxtaglomerular apparatus. An elevation in efferent renal sympathetic nerve activity triggers the activation of alpha 1 adrenoreceptors on the renal tubular epithelial cells, resulting in sodium reabsorption and volume expansion. Excessive activation of the efferent renal sympathetic system can stimulate the release of renin from the juxtaglomerular apparatus, leading to the activation of the renin-angiotensin-aldosterone system. This, as a result, generates angiotensin II and increases aldosterone production, ultimately causing a rise in blood pressure [3]. Renal sympathetic denervation is a minimally invasive procedure performed through femoral access, where the renal afferent and efferent nerves are ablated using a radiofrequency catheter. By delivering controlled and low-dose radiofrequency energy, the procedure effectively disrupts the nerve fibers located within the outer layer (adventitia) of the renal artery [2]. Moreover, conditions such as congestive heart failure, atrial fibrillation, sleep-breathing disorders, and diabetes mellitus are all linked to excessive sympathetic drive. This procedure can potentially provide multiple benefits, such as enhancing glycemic levels, addressing sleep apnea, managing arrhythmias, and reducing oxidative stress [2]. Sympathetic denervation not only aids in better blood pressure regulation but also improves the associated comorbidities and risk factors that contribute to hypertension. 

## Review

In early studies, renal denervation (RDN) was thought to be a promising approach to manage refractory HTN [1]. However, recent trials have failed to confirm significant blood pressure (BP) control after RDN as compared to a placebo procedure or standard medical therapy [1]. Some of the discrepancies can be attributed to the fact that earlier reports were uncontrolled studies that failed to include larger populations and assess compliance with medical therapy [2]. As a result, this analysis aims to explore the use of renal denervation as an alternative antihypertensive management in refractory hypertension. After analyzing the latest data, this review will serve to offer insight on the benefits and limitations of renal denervation as a therapeutic approach in the management of patients with refractory hypertension [2]. 

The inclusion criteria for this review included literature that evaluated the use of renal denervation procedures to control resistant HTN despite the use of combination antihypertensives at maximally tolerated doses. Age more than 18 years but less than 80 years of age. Systolic blood pressure (SBP) is more than 140 mmHg. Most of the data primarily originated from observational, randomized, blinded, and controlled studies. A search of PubMed and Google Scholar yielded several articles related to this topic, with exclusion criteria focusing on secondary hypertension caused by renal artery stenosis, primary aldosteronism, pheochromocytoma, or Cushing's syndrome. Also, renal anatomical abnormalities or medical conditions that make the renal denervation procedure unsafe or impractical, such as severe renal impairment like end-stage renal disease. A literature review of these cases was conducted, evaluating BP measurements after renal denervation as compared to the use of combination antihypertensive therapy or a sham procedure (Table 1). 

**Table 1:** Results of the literature review conducted. RDN: renal denervation, BP: blood pressure, HTN: hypertension, FDA: Federal Drug Administration.

Authorship and year of publication	Objective	Conclusion	Limitations
Bhatt et al. [1]	To determine if renal denervation provides more effective BP control than standard therapy in patients with resistant HTN.	Findings did not show a significant reduction in BP readings with RDN after six months when compared to sham control.	Medication adherence was not confirmed. A longer follow-up is needed. Lack of direct measurement to evaluate if renal nerves were in fact denervated. The findings are specific to the catheter used during the surgical procedure and cannot be applied to alternative denervation systems.
Coppolino et al. [2]	To evaluate the short- and long-term effects of RDN in individuals with resistant HTN.	Findings were inconclusive when determining if RDN provides a more effective BP control as compared to standard therapy and sham control. Bradycardia was noted to be a significant side effect of the procedure.	Studies were focused on a small population and short treatment periods.
Bravo et al. [3]	To determine if the denervation of renal sympathetic nerves provides a more efficient BP control than standard medical therapy.	Catheter-induced renal denervation decreased systemic BP for up to 12 months.	Lack of a control group. Medication modification took place during the study.
Davis et al. [4]	Determine the effectiveness and safety of renal denervation in resistant HTN.	RDN resulted in a substantial BP reduction after six months.	Observational studies are likely affected by confounding or selection bias. <70% follow-up rate at six months. Five different catheters were used, with no comparable data between the efficacy of each catheter.
Esler et al. [5]	To assess the effectiveness and safety of sympathetic RDN in BP control in resistant HTN.	Catheter-based renal denervation is safe and effective in the lowering of BP.	Follow-up of greater than six months is needed to evaluate the likelihood of sustained BP reduction.
Geisler et al. [6]	To assess the long-term clinical benefits of RDN in resistant HTN.	The catheter-based procedure may reduce cardiovascular morbidity and mortality in patients with resistant HTN.	Most participants were Caucasian.
Simplicity HTN1 Investigators [7]	To evaluate the durability and safety of BP reduction after RDN.	Renal sympathetic denervation appears to be a potentially useful and durable option in the management of refractory hypertension.	Lack of a control group. Limited number of participants reaching a 24-month follow-up. A modification of the antihypertensive regimen took place.
Kandzari et al. [8]	To evaluate the effectiveness and safety of bilateral catheter-based RDN for the treatment of uncontrolled HTN despite the use of multi-antihypertensive therapy. To address some of the limitations in the previously available studies.	Pending results will be submitted to US FDA for approval.	N/A
Krum et al. [9]	To assess safety and BP reduction effectiveness after RDN in patients with HTN secondary to sympathetic hyperactivity.	RDN causes substantial and sustained BP reduction, without serious adverse events, in patients with resistant hypertension.	Not a prospective control trial. BP changes achieved may have been confounded by medication changes during the study.
Messerli et al. [10]	Correlate Simplicity HTN-3 results to previous Simplicity HTN studies.	No significant BP reduction was noted after RDN when compared to previous studies.	N/A
Pappaccogli et al. [11]	To evaluate the effectiveness of RDN in controlled studies in comparison to a sham procedure or medical therapy.	RDN fails to show superiority to a sham procedure or medical therapy	N/A
Pisano et al. [12]	To evaluate the short- and long-term effects of RDN in resistant hypertension.	RDN might be effective in lowering BP in patients with resistant hypertension.	Sufficient data is not available. Longer follow-up periods are needed. A larger sample population is needed.
Prochnau et al. [13]	To analyze the effectiveness and safety of sympathetic RDN in the management of refractory HTN.	Preliminary results show that the use of RDN is a potential and safe procedure demonstrating a significant lowering of mean BP in comparison to baseline during short-term follow-up.	Long-term outcomes need to be further investigated.
Stouffer et al. [14]	To understand the role of sympathetic nerve interruption during renal denervation and its effect in BP.	Further studies are needed to determine the effectiveness of RDN.	Lack of control group. Need for restriction of medication modification during the study.
Azizi et al. [15]	To evaluate the effectiveness and safety of endovascular ultrasound RDN in patients with hypertension who are unresponsive to at least three antihypertensive drugs.	RDN reduced daytime ambulatory systolic blood pressure more than the sham procedure.	Short treatment period.

Clinical studies, such as Simplicity HTN-1, correlated renal denervation with a significant decrease in systolic BP [8]. Promising results then led to the conduction of the Simplicity HTN-2 trial. In this study, 190 patients were randomly allocated to renal denervation or control groups and assessed for improved BP control at six months. It was observed that BP measurements in the renal denervation group were reduced by 32 mmHg of systolic and 12 mmHg of diastolic from a baseline of 178/96 mmHg, whereas they did not significantly differ from the baseline in the control group. No serious procedure/device-related complications or adverse events were observed between groups [5]. In addition to the Simplicity trials, the Markov model was another study that concluded RDN effectively lowers BP readings in this patient population. The study analyzed a randomized control trial (RCT) for one month where participants had uncontrolled HTN with a SBP of more than 160 mmHg. Individuals in this study were taking an average of five blood pressure-lowering medications and had a mean age of 58. Results from this investigation indicated that RDN for resistant HTN was not only cost-effective when compared to other available medical treatments, but it also substantially reduced the risk for cardiovascular and end-stage renal disease. Additionally, the procedure reduced SBP by 32±23 mmHg from 178±18 mmHg at baseline on standard-of-care treatment [6]. 

Moreover, a systematic review and meta-analysis conducted in 2013 also described promising results to support the use of RDN in refractory HTN. Thirteen studies were reviewed, including two randomized control trials (RCTs), one observational study with a control group, and nine observational studies without a control group. It was observed that RDN resulted in better BP control in this patient population, with a greater reduction in mean systolic and diastolic readings at six months in the controlled studies as compared to medically treated patients. However, it is important to note that most of the included studies were observational, and the results may have been affected by confounding or selection bias. Additionally, five different catheters were available for the RDN procedure, but there was insufficient data to compare the efficacy of the different catheters [4]. Analogously, a review of 15 studies involved over 1400 participants with resistant HTN who were followed from three to 24 months. Although results were inconclusive on determining whether RDN improves cardiovascular and renal complications associated with persistently elevated blood pressures, the study supports the use of the procedure as an alternative therapeutic management when compared to other treatments or no treatment [12]. Preliminary results in another trial also indicated a lowering in BP readings after denervation. Although this study showed promising results, only 12 patients were enrolled, and postprocedural results were compared to baseline [13]. To achieve greater statistical significance, it is crucial to analyze a larger population and include a control group for comparison.

Another promising study evaluated the efficacy of RDN at 24 months. Since sympathetic nerves have the potential to grow, this study focused on the durability of BP control after renal nerve damage. In this analysis, 153 patients meeting the same inclusion criteria as previous studies described above were enrolled. About 92% of patients had a postprocedural 10 mmHg BP reduction with little to no change in the number of antihypertensive medications compared to baseline. Furthermore, the magnitude of BP reduction was found to be greater than or equal to the BP control seen at 12 months. This suggests a possible override of renal reinnervation and vascular remodeling, leading to persistent BP-lowering effects over the two-year postprocedural period. Limitations included no control group for comparison of BP response over time, and only a limited number of patients reached the 24-month follow-up period. Changes in antihypertension medication were also permitted during the study. However, these limitations were taken into consideration and addressed in a follow-up trial previously discussed (Simplicity HTN-2) [7]. Another non-randomized study where participants were followed for one year had similar findings. Fifty patients with persistent HTN despite combination antihypertensives were monitored to assess the effectiveness of RDN; 45 of the participants received percutaneous radiofrequency catheter-based therapy, and five were not eligible due to anatomical exclusions. Patients who underwent treatment showed a noticeable and lasting decrease in blood pressure, while those who were not eligible experienced an increase in average blood pressure readings. The effectiveness of renal denervation was also evaluated by examining the levels of noradrenaline release from the kidneys before and after the procedure. Patients who had successful blood pressure control after renal denervation showed a significant reduction in noradrenaline release. Furthermore, the absence of a decrease in the achieved blood pressure reduction indicated that there was no nerve fiber recovery, regrowth, or development one year after the procedure [9]. 

In comparison, a prospective, single-blinded, randomized controlled trial (RCT) enrolled a total of 530 patients with resistant primary hypertension. The primary focus was to evaluate the change in systolic blood pressure at six months, while considering safety including cause of death, end-stage renal disease, embolic events leading to end-organ damage, renovascular complications, hypertensive crisis at one month, and new renal artery stenosis >70% at six months. Candidates were on maximum tolerated doses of at least three antihypertensives, including a diuretic, with an initial systolic blood pressure of 160 mmHg or higher. Exclusion criteria included secondary hypertension, including anatomical causes (i.e., renal artery stenosis of >50%, renal artery aneurysm), and more than one hospitalization in the previous year due to a hypertensive emergency. The treatment group underwent renal artery denervation with the use of radiofrequency energy, while the sham control only had renal angiography. While renal denervation appeared to be safe in this trial, a significant effect on systolic blood pressure was not observed. The need for antihypertensives was like that at baseline after the denervation and sham procedure, with no significant between-group difference in blood pressure at six months. However, this study was limited by its ability to confirm medication adherence and the lack of a direct measure to confirm the successful denervation of renal nerves. Additionally, these findings contradicted previous clinical data. Discrepancies may be a result of prior studies being non-randomized trials that lacked blinding and standardization of medication regimens. Previous studies also failed to include a control group and compared treatment results with baseline observations [1,14]. To overcome these limitations, the Simplicity HTN-3 trial was designed to include a sham procedure and subject participants to blinding randomization. Furthermore, the study ensured equal management of groups to uphold blinding, maintenance of baseline antihypertensive regimens between groups without changes for six months, and patient follow-up for three years. Despite these measures, a significant reduction in systolic BP was not observed, challenging the promising findings of the previous two Simplicity studies [10].

Similarly, a systematic review analyzing twelve different RCTs on this topic had comparable observations. A total of 1149 adults were assigned to undergo renal denervation and followed for three to 12 months. BP was monitored during this time frame, and results were compared to those of patients undergoing a sham procedure or on standard medical therapy. Inclusion criteria involved adults older than 18 years of age with refractory HTN and a BP of >140/90 mmHg or 130/80 mmHg in individuals with type II diabetes mellitus; participants were also on concomitant use of at least three antihypertensive drugs, including a diuretic. Overall, there was no evidence of renal denervation resulting in significant BP control or decreased cardiovascular morbidity or mortality when compared to standard treatment. Although renal function was not affected by the procedure, it was noted that patients undergoing renal denervation were at increased risk of episodes of bradycardia. One of the studies observed a significant reduction in heart rate (HR) after six months of the procedure. These changes did not correlate with BP measurements at three- and six-months post-RDN, so it's postulated that RDN decreases HR independently to its BP-lowering effects [3]. Furthermore, a meta-analysis of 11 publications also failed to find statistical significance in BP improvement after the use of RDN. The article reviewed three double-blinded RCTs with a sham control and eight open-label studies where treatment with medical therapy served as a control, but RDN did not prove superiority to alternatives as previously described in earlier publications [11]. 

Additionally, the RADIANCE-HTN TRIO trial determined that compared to the sham procedure, RDN resulted in a greater reduction in daytime systolic blood pressure after a two-month follow-up. This multinational study enrolled 989 patients who met specific criteria: ages ranging from 18 to 75 years, absence of comorbidities associated with secondary hypertension such as diabetes or obstructive sleep apnea, glomerular filtration rate (GFR) above 40 ml/min/m^2^, and normal renal artery anatomy. All participants were on a combination of three or more antihypertensive medications [15]. The average age of the patients was 53 years, with 20% being women and 20% being of black ethnicity. At baseline, their blood pressure was measured as 163/104 mmHg despite taking more than four antihypertensive medications [15]. Before randomization into either the simulated procedure or RDN, patients were switched to a once-daily antihypertensive medication, and their daytime ambulatory blood pressure had to be above 135/85 mmHg. The findings of the study revealed that after the two-month follow-up, the RDN group experienced an 8 mmHg decrease in blood pressure compared to a 3 mmHg decrease in the sham procedure group. The study's limitation is the short monitoring duration, which doesn't provide insight into whether the lowered blood pressure is sustained in the long term. While RDN shows potential as an alternative therapy for blood pressure control, more extensive data collection is still necessary to assess its long-term efficacy and safety. 

## Conclusions

Hypertension remains a global public health concern as it is one of the leading risk factors for the development of cardiovascular disease. Although multidrug therapy has been widely used in the management of treatment-resistant HTN, BP control in this patient population has been suboptimal. Several studies reported encouraging results with the use of an innovative renal denervation approach. However, the use of this procedure in patients with refractory HTN despite multiple antihypertensive therapies at maximal dose remains controversial given the short duration of monitoring in several studies. Newer studies analyzing larger populations with the inclusion of control groups and standardization of therapy have failed to reproduce the previously reported benefits of RDN. Although this innovative procedure may be a promising therapeutic management for refractory HTN, further investigation is still needed. New studies should focus on sampling a larger number of participants and longer follow-up periods to obtain better insight on the effects of renal denervation in patients with refractory HTN while incorporating side effects and safety. 
